# Impact of Obstructive Sleep Apnea Diagnosed Using the STOP-Bang Questionnaire Scale on Postoperative Complications Following Major Cardiac Surgery: A Prospective Observational Cohort Study

**DOI:** 10.7759/cureus.26102

**Published:** 2022-06-20

**Authors:** Fatemeh Javaherforooshzadeh, Mohammadreza Amjadzadeh, Habib Haybar, Amir Sharafkhaneh

**Affiliations:** 1 Department of Anesthesia, Ahvaz Anesthesiology and Pain Research Center, Ahvaz Jundishapur University of Medical Sciences, Ahvaz, IRN; 2 Department of Radiology, Ahvaz Anesthesiology and Pain Research Center, Ahvaz Jundishapur University of Medical Sciences, Ahvaz, IRN; 3 Department of Cardiology, Atherosclerosis Research Center, Ahvaz Jundishapur University of Medical Sciences, Ahvaz, IRN; 4 Department of Medicine, Baylor College of Medicine, Houston, USA

**Keywords:** sleep disordered breathing, stop-bang questionnaire, obstructive sleep apnea, coronary artery disease, cardiac surgery

## Abstract

Purpose

Obstructive sleep apnea (OSA) is a common and often undiagnosed condition in patients undergoing major surgeries, including cardiac surgery. This disorder is associated with peri- and postoperative problems. This study measured the association between OSA and peri- and postoperative complications in patients undergoing elective cardiac surgery.

Methods

Candidates for elective cardiac surgery were evaluated for OSA by the STOP-Bang questionnaire before the surgery. We evaluated patients before and after the operation regarding the cardiac, respiratory, and neurologic complications. We divided the participants into high-risk (score of 5-8), intermediate-risk (score of 3-4), and low-risk groups (score of 0-2) based on the STOP-Bang questionnaire.

Results

Of the 306 patients who underwent cardiac surgery, 173 (56.5%) were in the high-risk group, 100 (32.7%) were in the intermediate-risk group, and 33 (10.8%) were in the low-risk group for OSA.

Patients in the high-risk group were significantly older than the other two groups (p value=0.013), had higher BMI (p<0.001), and suffered more from relevant comorbid conditions, including diabetes mellitus, hypertension, and hyperlipidemia (all p-values significant at < 0.05). However, not significant, patients in the high-risk group suffered more from postoperative complications including cardiac, respiratory, and neurological complications.

Conclusion

OSA is common in patients undergoing cardiac surgery. Our findings indicate that these patients manifest a higher incidence of postoperative complications compared to those with a lower risk of OSA. Because of the limited use of polysomnography, a simple STOP-Bang questionnaire is beneficial to screen patients for the risk of OSA peri-operatively, and patients diagnosed with OSA can get extra care during and after the surgery.

## Introduction

Sleep-disordered breathing (SDB) is a common disease, with a prevalence of approximately 13% in the male and 6% in the female population in the age range of 30-70 years [1]. Previous studies have shown associations between SDB and many underlying diseases, such as hypertension (HTN), diabetes mellitus (DM), coronary artery disease, and heart failure (HF). SDB has three subtypes: central sleep apnea (CSA), obstructive sleep apnea (OSA), and mixed sleep apnea. OSA is characterized by episodes of complete or partial airway obstruction. The prevalence of moderate-to-severe OSA in the general adult population ranges from 6% to 17% and is higher in men, obese, and elderly people, though most of them remain undiagnosed. The prevalence of OSA is even higher in people with underlying cardiovascular diseases [2-5].

The effect of undiagnosed OSA on postoperative complications has not been well studied. A prospective cohort study by Ambrosii et al. on patients undergoing operations other than cardiac surgery suggests that patients with OSA are at greater risk for postoperative cardiovascular and respiratory complications [6]. In patients undergoing cardiac surgery, more than half of them are identified to have non-treated OSA [7].

Many previous studies were limited to patients undergoing coronary artery bypass grafting (CABG) [8-11], which restricted the comprehensiveness of their findings.

Many patients who undergo different surgeries do not receive a confirmed diagnosis for OSA preoperatively. Hence, a guideline published by the American Society of Anesthesiologists recommends screening the patients for risk of OSA before the surgery [12].

However, polysomnography (PSG) is the gold standard test for the diagnosis of OSA. It is an expensive test that is not generally available and requires highly-trained personnel to perform it. It also requires an entire night of recording, which may delay the surgery. Therefore, it is crucial to measure the risk of OSA using a validated screening tool preoperatively. For this purpose, there are screening tools that are more available and easy to use, such as the STOP-Bang questionnaire or Berlin Questionnaire.

STOP-Bang questionnaire is a widely used tool for screening OSA patients with a sensitivity of 93% in detecting patients with moderate-to-severe sleep apnea, and a sensitivity of 100% in detecting patients with severe sleep apnea [13]. However, STOP-Bang showed a low specificity (36%) for OSA [14]. There may be many false-positive cases of OSA.

This study examined the hypothesis that patients undergoing elective cardiac surgery who are at a greater risk for OSA assessed by the STOP-Bang questionnaire have an increased risk of developing postoperative complications, including cardiac, respiratory, and neurological complications, increased length of stay (LoS) in the intensive care unit (ICU), and death.

## Materials and methods

This prospective cohort study was conducted in Golestan Hospital, Ahvaz, Iran, from February 2020 to January 2021, with ethics code No. IR.AJUMS.REC.1398.595 received from the Anesthesiology and Pain Research Center, Ahvaz Jundishapur University of Medical Sciences, Ahvaz, Iran.

This study evaluated the association between the risk of OSA assessed by the STOP-Bang questionnaire and postoperative complications in patients undergoing elective cardiac surgery. The inclusion criteria were all adult patients (age ≥ 18) who were scheduled for elective cardiac surgery. The exclusion criteria included patient refusal, history of OSA, and history of tracheostomy.

A total of 306 patients met the inclusion and exclusion criteria and were included in the study. Eligible patients provided written informed consent. They were evaluated by the use of the STOP-Bang questionnaire the night before surgery. All the baseline characteristics and demographic parameters were recorded based on the patients' self-reported data and the data retrieved from medical records. Additionally, we recorded all the adverse events and complications for each group of patients during and after the surgery until hospital discharge.

The primary outcomes were postoperative complications (cardiac, pulmonary, neurologic, and general) observed during the hospital stay (about 10 days). Cardiac complications included any of the following: (1) HTN and hypotension, (2) arrhythmia, (3) cardiac arrest, and (4) myocardial infarction (MI), confirmed by an elevated troponin blood test in addition to the presence of clinical evidence of ischemia. Respiratory complications included any of the following: (1) prolonged mechanical ventilation, defined as mechanical ventilation of more than 48 hours postoperatively, (2) pneumonia, (3) atelectasis, (4) need for tracheostomy, (5) hypoxemia was considered if the patient had oxygen desaturations less than 90% or a reduction of 4% or more from the last recorded value, and (6) need for reintubation. Neurological complications included any of the following: (1) clinically diagnosed encephalopathy and (2) delirium. General complications included ICU readmission, fever, wound infection, and in-hospital death.

Secondary outcomes were considered any other adverse events that occurred during the 30-day period after discharge. For this purpose, patients were evaluated through a telephone encounter for readmission and death due to any postoperative complication.

Statistical analysis

Numbers and percentages were used to express categorical variables, and mean ± standard deviation was used to express continuous variables. To compare variables regarding patient characteristics and postoperative complications in the three patient groups, we used the chi-square test for categorical variables and the one-way ANOVA (analysis of variance) for continuous variables. Linear regression analyses were conducted to predict the impact of independent variables such as OSA, age, body mass index (BMI), left ventricular ejection fraction (LVEF), DM, and HTN on ICU LoS and postoperative cardiac, respiratory, and neurological complications. SPSS software (Version 22.0, IBM Corp. Armonk, NY) and GraphPad Prism (Version 8.0.2) were used for statistical analysis.

## Results

Baseline characteristics of the groups

A total of 306 patients met the inclusion and exclusion criteria and were included in the study. According to the STOP-Bang questionnaire, patients were divided into three groups; low-risk (n=33), intermediate-risk (n=100), and high-risk (n=173). The baseline characteristics of all patients are classified in Table 1 based on their risk for OSA. The mean age of the patients was 59.1 ± 9.8 years, and patients in the high-risk group were significantly older than the other two groups. Also, patients in the high-risk group had a higher BMI. They were also significantly more prone to suffer from DM, HTN, and HLP. However, not significant, patients in the high-risk group for OSA were more often diagnosed with HF and more often had a history of MI and transient ischemic attack/cerebrovascular accident (TIA/CVA) in the past. The drug history of patients in all three groups is shown in Table 1. Patients in the high-risk group had greater use of medications, such as antihypertensive drugs, diuretics, and statins, compared to the other two groups. We did not find any significant differences between the three patient groups in terms of glomerular filtration rate, hypothyroidism, and pulmonary HTN.

**Table 1:** Baseline Characteristics of the Study Population BMI, body mass index (kg/m^2^); HTN, hypertension; MI, myocardial infarction; HF, heart failure; TIA/CVA, transient ischemic attack/cerebrovascular accident; COPD, chronic obstructive pulmonary disease; DM, diabetes mellitus; GFR, glomerular filtration rate (mL/min/1.73 m^2^) *Statistically significant.

Characteristics	Low Risk, 33 (10.8%)	Intermediate Risk, 100 (32.7%)	High Risk, 173 (56.5%)	p-Value
Demographics				
Age	54.5±12.9	59.1±10.2	60.1±8.6	0.013*
Sex (male)	8 (24.2)	62 (62)	129 (74.6)	<0.001*
BMI	27.1±3.6	27.1±4.2	29.5±4.8	<0.001*
Baseline characteristics				
HTN	20 (60.6)	76 (76)	163 (94.2)	<0.001*
Pulmonary HTN	0 (0.0)	2 (2)	0 (0.0)	-
Previous MI	9 (27.3)	38 (38)	70 (40.5)	0.360
HF	12 (36.4)	49 (49)	76 (43.9)	0.424
TIA/CVA	4 (12.1)	9 (9)	16 (9.2)	0.858
COPD	1 (3.0)	16 (16)	27 (15.6)	0.130
DM	14 (42.4)	46 (46)	103 (59.5)	0.041*
Hyperlipidemia	18 (54.5)	60 (60)	140 (80.9)	<0.001*
Hypothyroidism	0 (0.0)	2 (2)	2 (1.2)	0.763
Smoking	5 (15.2)	37 (37)	75 (43.4)	0.009*
Creatinine (mg/dL)	1.0±0.2	1.2±0.8	1.2±0.3	0.306
GFR	72.5±21.6	74.5±29.5	78.5±26.0	0.330
Drug history				
Beta-blockers	19 (57.6)	67 (67.0)	111 (64.2)	0.616
Calcium channel blockers	2 (6.1)	15 (15.0)	43 (24.9)	0.016*
Angiotensin II receptor blockers	10 (30.3)	37 (37.0)	93 (53.8)	0.005*
Angiotensin-converting enzyme inhibitors	4 (12.1)	16 (16.0)	27 (15.6)	0.858
Diuretics	11 (33.3)	31 (31.0)	67 (38.7)	0.420
Statins	14 (42.4)	71 (71.0)	146 (84.4)	<0.001*
Aspirin	20 (60.6)	81 (81.0)	145 (83.8)	0.009*
Clopidogrel	16 (48.5)	51 (51.0)	90 (52.0)	0.930
Nitrocontin	8 (24.2)	42 (42.0)	86 (49.7)	0.022*

Patients in all three groups were evaluated for coronary artery disease based on the number of involved vessels (one-, two-, and three-vessel). Our high-risk group had a higher number of patients with one- and three-vessel involvement compared to the other two groups (p<0.05). However, not significant, patients in the high-risk group were more prone to have aortic valve stenosis, aortic valve insufficiency, pulmonary valve insufficiency, mitral valve regurgitation, and tricuspid valve regurgitation. We found no significant difference between our studied groups regarding LVEF (Table 2).

**Table 2:** Angiographic and Echocardiographic Characteristics of the Study Population LVEF, left ventricular ejection fraction (%) *Statistically significant.

Characteristics	Low Risk, 33 (10.8%)	Intermediate Risk, 100 (32.7%)	High Risk, 173 (56.5%)	p-Value
One-vessel disease	0 (0.0)	10 (10.0)	0 (0.0)	<0.001*
Two-vessel disease	4 (12.1)	10 (10.0)	18 (10.4)	0.942
Three-vessel disease	20 (60.6)	72 (72.0)	151 (87.3)	<0.001*
LVEF	43.9±9.6	42.7±10.9	42.5±11.4	0.808
Aortic valve stenosis	0 (0.0)	4 (4.0)	8 (4.6)	0.455
Aortic valve insufficiency	12 (34.6)	31 (31.0)	62 (35.8)	0.695
Pulmonary valve insufficiency	13 (39.4)	42 (42.0)	77 (44.5)	0.829
Mitral valve Insufficiency	0 (0.0)	2 (2.0)	0 (0.0)	0.188
Mitral valve stenosis	5 (15.2)	2 (2.0)	2 (1.2)	0.001
Mitral valve regurgitation	27 (81.8)	83 (83.0)	135 (78.0)	0.592
Tricuspid valve regurgitation	24 (72.7)	69 (69.0)	126 (72.8)	0.786

Intraoperative outcomes

All cardiac surgeries performed on patients are listed in Table 3. It was found that the number of patients who underwent CABG surgery was significantly higher in the high-risk group compared to the other two groups. During operation, complications such as difficult intubation, hypoxemia, arrhythmia, and HTN were assessed in all three patient groups. However, not significant, patients in the intermediate-risk and high-risk groups were at higher risk for difficult intubation. The number of patients who had hypoxemia, HTN, and arrhythmia during the operation was greater in the high-risk group compared to the other two groups, but none of these differences were statistically significant.

**Table 3:** Intraoperative Data of the Study Population CABG, coronary artery bypass grafting; ASD, atrial septal defect; VSD, ventricular septal defect; HTN, hypertension *Statistically significant.

Characteristics	Low Risk, 33 (10.8%)	Intermediate Risk, 100 (32.7%)	High Risk, 173 (56.5%)	p-Value
Type of operation				
CABG only	22 (66.6)	90 (90.0)	157 (90.8)	<0.001*
Valve repair only	0 (0.0)	0 (0.0)	2 (1.2)	0.461
Valve replacement only	7 (21.2)	4 (4.0)	2 (1.2)	<0.001*
ASD repair only	2 (6.1)	2 (2.0)	0 (0.0)	0.015*
VSD repair only	0 (0.0)	2 (2.0)	0 (0.0)	0.126
CABG/valve replacement/ ASD repair	2 (6.1)	0 (0.0)	0 (0.0)	<0.001*
CABG/valve repair	0 (0.0)	1 (1.0)	4 (2.3)	0.806
CABG/valve replacement	0 (0.0)	1 (1.0)	6 (3.5)	0.435
Valve repair/ASD repair	0 (0.0)	0 (0.0)	1 (0.6)	>0.999
Valve repair/valve replacement	0 (0.0)	0 (0.0)	1 (0.6)	>0.999
Intraoperative outcomes				
Difficult intubation	0 (0.0)	9 (9.0)	8 (4.6)	0.109
Hypoxemia	1.1±0.2	1.0±0.2	1.1±0.3	0.246
Arrhythmia	0(0.0)	2(2.0)	4 (2.3)	>0.999
HTN	7 (21.2)	30 (30.0)	56 (32.4)	0.440
Death	0 (0.0)	0 (0.0)	0 (0.0)	-

Postoperative outcomes

Respiratory Complications

It was found that hypoxemia was more frequent in patients in the high-risk group compared to the other two groups, but the difference was not statistically significant. Although more patients in the high-risk group needed prolonged use of mechanical ventilation, this difference was not statistically significant. Also, the reintubation rate was higher in the high-risk and intermediate-risk groups compared to the low-risk group, but the difference was not statistically significant. Atelectasis and pneumonia were two complications that only manifested in the high-risk group (Table 4).

**Table 4:** Postoperative Outcomes of the Study Population AF, atrial fibrillation; HTN, hypertension; MI, myocardial infarction; ICU, intensive care unit; LoS, length of stay *Statistically significant.

Characteristics	Low Risk, 33 (10.8%)	Intermediate Risk, 100 (32.7%)	High Risk, 173 (56.5%)	p-Value
Respiratory complications				
Prolonged mechanical ventilation	0 (0.0)	1 (1.0)	2 (1.2)	>0.999
Atelectasis	0 (0.0)	0 (0.0)	2 (1.2)	-
Pneumonia	0 (0.0)	0 (0.0)	4 (2.3)	0.421
Tracheostomy	0 (0.0)	0 (0.0)	0 (0.0)	-
Reintubation	1 (3.0)	4 (4.0)	4 (2.3)	0.611
Hypoxemia	4 (12.1)	14 (14.0)	26 (15.0)	0.943
Cardiac complications				
AF	5 (15.2)	12 (12.0)	12 (6.9)	0.194
Bradycardia	0 (0.0)	3 (3.0)	5 (2.9)	0.886
Tachycardia	2 (6.1)	5 (5.0)	3 (1.7)	0.174
HTN	2 (6.1)	21 (21.0)	61 (35.3)	<0.001*
Cardiac arrest	1 (3.0)	1 (1.0)	5 (2.9)	0.521
MI	0 (0.0)	0 (0.0)	0 (0.0)	-
Neurological complications				
Encephalopathy	0 (0.0)	0 (0.0)	0 (0.0)	-
Delirium	2 (6.1)	2 (2.0)	4 (2.3)	0.355
General complications				
Weaning time	878.6±484.9	826.1±672.7	1061.1±2741.1	0.656
Fever	1 (3.0)	0 (0.0)	6 (3.5)	0.133
Wound infection	2 (6.1)	0 (0.0)	0 (0.0)	0.011*
ICU readmission	4 (12.1)	2 (2.0)	2 (1.2)	0.006*
ICU LoS (day)	3.0±1.5	2.9±1.2	2.9±1.3	0.975
Postoperation death	1 (3.0)	1 (1.0)	5 (2.9)	0.521
Insulin infusion in ICU	12 (36.4)	44 (44.0)	94 (54.3)	0.079

Cardiac Complications

The number of patients with atrial fibrillation (AF) was higher in the high-risk and intermediate-risk groups compared to the low-risk group, but the difference was not statistically significant. Additionally, the number of patients with postsurgical HTN was higher in the high-risk group compared to the other two groups, which was statistically significant. Moreover, patients in the high-risk group were more susceptible to developing cardiac arrest and bradycardia after the surgery, but no significant differences were observed between the three patient groups (Table 4).

Neurological Complications

According to the neurological complications, the results showed that none of the patients had encephalopathy. However, the number of patients with delirium was higher in the high-risk group compared to the other two groups, but the difference was not statistically significant (Table 4).

General Complications

The number of non-diabetic patients who received insulin infusion in the ICU was higher in the high-risk group compared to the other two groups, but the difference was not statistically significant. However, patients in the high-risk group were more prone to develop a fever after the surgery, but the difference was not statistically significant. Although it was not statistically significant, we recorded more in-hospital death in the high-risk group compared to the other two groups. The analyses did not confirm significant differences between the three patient groups regarding weaning time and ICU LoS. Unexpectedly, we significantly recorded more ICU readmission and wound infection cases in the low-risk group than in the other two groups (Table 4).

Predictors of postoperative complications

Based on linear regression analysis, it was found that age and DM are independent predictors of postoperative respiratory complications. It was found that male sex and OSA are independent predictors of postoperative cardiac complications. Additionally, it demonstrated that LVEF<50% is an independent predictor of postoperative respiratory, cardiac, and neurological complications. Linear regression analysis showed that postoperative ICU LoS is significantly dependent on age, LVEF<50%, and CABG surgery (Table 5).

**Table 5:** Predictors for Respiratory, Cardiac, and Neurological Complications, and ICU LoS ICU, intensive care unit; LoS, length of stay; OSA, obstructive sleep apnea; HTN, hypertension; DM, diabetes mellitus; LVEF, left ventricular ejection fraction (%); BMI, body mass index (kg/m^2^); CABG, coronary artery bypass grafting Values are presented as standardized beta coefficients. *Statistically significant.

Postoperative Variables	Respiratory (Model I)		Cardiac (Model II)		Neurological (Model III)		ICU LoS (Model IV)	
Independent variable	Beta	p-Value	Beta	p-Value	Beta	p-Value	Beta	p-Value
OSA	0.022	0.704	0.184	0.002*	0.018	0.750	0.040	0.467
Male sex	-0.053	0.352	-0.133	0.026*	0.020	0.728	-0.019	0.719
Age	0.176	0.002*	0.080	0.163	0.104	0.067	0.162	0.003*
HTN	-0.066	0.245	0.057-	0.363	0.080	0.161	-0.024	0.666
DM	-0.161	0.005*	0.001	0.984	-0.016	0.781	0.007	0.896
LVEF<50%	0.123	0.029*	0.123	0.030*	0.150	0.008*	0.244	<0.001*
BMI ≥ 30	0.105	0.067	0.086	0.163	0.097	0.094	0.010	0.853
CABG	-0.102	0.084	0.041-	0.472	0.058	0.311	0.313-	<0.001*

Postoperative follow-up

Thirty days after discharge, patients were evaluated through a telephone encounter for readmission and death due to any postoperative complication. It was found that significantly 17 patients in the high-risk group and 12 patients in the other two groups (six patients in each group) were readmitted to the hospital prior to day 30 after their discharge. Additionally, no postdischarge death was recorded in any patient group.

## Discussion

Our data showed that HTN occurs more frequently in the high-risk group in the postoperative period. Unexpectedly, the low-risk group had more ICU readmission and wound infection. The hospital readmission rate was higher in the high-risk OSA group compared to the other two groups.

The present study confirms the previous findings of the high prevalence of OSA by using the STOP-Bang questionnaire in patients undergoing cardiac surgery. Because of the increased rate of HF and metabolic syndrome, which are linked to OSA, patients undergoing cardiac surgery have a higher risk of OSA [15,16]. Our findings are in concordance with previous studies [7,11]. The difference in the prevalence of OSA in these studies might be due to different definitions and diagnostic tools that were used for detecting OSA.

Patients in the high-risk group for OSA were significantly more prone to develop postoperative HTN. In a cohort study by Liao et al., patients with OSA were more likely to develop HTN postoperatively (they did not reach statistical significance) [17].

The relationship between OSA and AF is well-documented in the literature [18]. Furthermore, OSA is an independent risk factor of postoperative AF in cardiac surgery [19]. In our study, the number of patients with AF was higher in the high-risk and intermediate-risk OSA groups compared to the low-risk group. Similar to our findings, Mason et al. reported that patients with sleep apnea undergoing major cardiac surgery are more prone to have postoperative cardiovascular complications [20]. Van Oosten et al. and Mungan et al. demonstrated in their studies that patients with a higher risk of OSA undergoing CABG developed more postoperative AF [10,21].

However, the rate of bradycardia and cardiac arrest was higher in patients in our high-risk OSA group but did not reach statistical significance.

The correlation between OSA and postoperative cardiac surgery complication rates is a matter of controversy. Although some studies reported a correlation between OSA and increased risk of postoperative cardiac complications, others did not find any significant association between OSA and postoperative cardiac complications [9,22].

Our data showed that OSA, male sex, and LVEF<50% are independent variables associated with increased risk of postoperative cardiac complications. There was one study that stated patients with OSA had a significantly lower rate of MI compared to those without OSA [23].

A systematic review study by Vasu et al. demonstrated that drugs such as sedatives, anesthetics, and opioids, which are administered during the intraoperative and postoperative periods, may result in increasing the collapsibility of the upper respiratory airway, reduced hypoxic and hypercapnic ventilatory response, and an increased threshold for arousal from sleep, which ends in worsening of OSA and may increase the risk of unfavorable postoperative complications [24].

Thus, monitoring and maintaining vital signs during anesthesia defends the patient from postoperative adverse events. However, the association between OSA and postoperative respiratory complications is a matter of debate. Tafelmeier et al. demonstrated that the need for postoperative tracheostomy was significantly higher in patients with OSA. However, they stated that the occurrence of respiratory complications such as acute respiratory distress syndrome and pneumonia was the same between patients with OSA and patients without SDB [9]. Mokhlesi et al. reported that patients with OSA undergoing cardiac surgery had an increased risk of postoperative respiratory complications such as respiratory failure, pneumonia, reintubation, and tracheostomy compared to those without OSA [25]. Another study by Rupprecht et al. showed that the frequency of postoperative respiratory complications was higher in patients with moderate-to-severe OSA compared to those with no OSA [11].

In contrast, Gali et al. reported that they did not find any differences between OSA and non-OSA groups in case of reintubation, prolonged mechanical ventilation, and pneumonia, but they stated that patients with OSA had significantly more postoperative ventilation hours compared to non-OSA patients [26].

This study also evaluated patients in case of respiratory complications; however, there was a trend toward more respiratory complications in patients with a higher risk of OSA as noted in the increased rate of reintubation, pneumonia, atelectasis, and hypoxemia, but it did not reach statistical significance. As reported by others, in our study, postoperative respiratory complications were associated with many independent factors, including age, DM, and LVEF<50% [7,8,10,19,20,27].

Additionally, this study assessed the relationship between OSA and postoperative neurological complications, including encephalopathy and delirium. None of our studied patients developed encephalopathy. Our data showed more patients in the high-risk OSA group developed delirium, but the difference did not reach statistical significance. Linear regression showed that LVEF<50% is an independent risk factor that increases the risk of postoperative neurological complications. Our results are consistent with the existing literature [7,27]. Roggenbach et al. showed that OSA might be a risk factor for postcardiac surgery delirium [28].

According to our initial hypothesis, we found an association between the higher risk of OSA and the postoperative increased rate of ICU readmission and wound infection. Prior studies demonstrated an increased risk of prolonged ICU and hospital LoS in patients undergoing major cardiac surgery [9,26,29]. Age, LVEF<50%, and CABG surgery are three independent risk factors for increased LoS in ICU.

It is important to note that the present evidence relies on the hospital's local policies, and ICU discharge is a multifactorial process that depends on many factors such as the number of beds available in the ICU or the general condition of patients. A previous study reported that institutional policies of ICU discharge affect the LoS in the ICU [30].

However, not significant, patients with an increased risk of OSA were more likely to have postoperative in-hospital death. A similar pattern of results was reported in other studies [7,11].

There was one study that stated that patients with OSA had a lower mortality rate compared to those without OSA [23].

Our findings demonstrated that the readmission rate in patients in the high-risk group is significantly higher compared to the other two groups, which is consistent with existing literature. Feng et al. reported that patients with OSA were more prone to readmission to the hospital 30 days after discharge. Gali et al. reported that the 30-day hospital readmission rate was higher in patients with known OSA compared to those without OSA [23,26].

Limitations

This study was a single-center investigation analysis, and most of the patients who underwent elective cardiac surgery were admitted to the hospital on the day of surgery; thus, we could not confirm the diagnosis of OSA by overnight PSG. It is obvious that questionnaires such as STOP-Bang, despite their simplicity and ease of use, are screening tools that aim to identify the risk of OSA in patients, but they do not establish the definitive diagnosis and severity of OSA. Also, the short duration of postdischarge follow-up and determination of 30-day outcomes through a telephone encounter were the other limitations.

## Conclusions

Our data showed that patients undergoing cardiac surgery are at a greater risk of undiagnosed OSA, which affects them in terms of intraoperative and postoperative complications. Because of the limited use of PSG, a simple STOP-Bang questionnaire can be used to screen patients for the risk of OSA preoperatively, and patients diagnosed with OSA can get extra care during and after the surgery. Also, further research is needed to find better methods for taking care of patients undergoing cardiac surgery who are at greater risk for OSA.
